# Comparative Analysis of Atherogenic Dyslipidemia Patterns in Alcoholic Versus Metabolic Dysfunction-Associated Steatotic Liver Disease: Implications for Cardiovascular Risk Stratification

**DOI:** 10.7759/cureus.108635

**Published:** 2026-05-11

**Authors:** Sushant Dhanavade, Mandakini Kshirsagar, Axita Vani

**Affiliations:** 1 Department of Biochemistry, Krishna Institute of Medical Sciences, Krishna Vishwa Vidyapeeth (Deemed to be University), Karad, IND

**Keywords:** afld, alcohol-related fatty liver disease, atherogenic profile, cardiovascular risk, dyslipidemia, lipid metabolism, masld, metabolic dysfunction-associated steatotic liver disease

## Abstract

Background: Abnormal lipid metabolism is a hallmark of both alcohol-related fatty liver disease (AFLD) and metabolic dysfunction-associated steatotic liver disease (MASLD), both of which heighten cardiovascular risk. However, comparative data on lipid abnormalities between these two conditions remain sparse, especially among South Asian populations.

Objective: To systematically compare atherogenic lipid profiles between patients with AFLD and MASLD.

Methods: This cross-sectional study enrolled 90 participants, including 30 with AFLD, 30 with MASLD, and 30 healthy controls. Fasting serum samples were assessed for total cholesterol (TC), low-density lipoprotein cholesterol (LDL-C), high-density lipoprotein cholesterol (HDL-C), triglycerides (TG), and non-HDL-C. Additionally, atherogenic indices and lipid ratios were calculated. Group differences were analyzed using ANOVA with post-hoc tests and multivariable regression (α = 0.05).

Results: Both disease groups showed significant dyslipidemia relative to controls (p < 0.001). MASLD patients exhibited the most unfavorable profile (TC = 220 ± 25 mg/dL, LDL-C = 150 ± 20 mg/dL, TG = 200 ± 50 mg/dL) compared to AFLD (TC = 200 ± 30, LDL-C = 130 ± 25, TG = 180 ± 60 mg/dL) and controls (TC = 180 ± 20, LDL-C = 110 ± 15, TG = 120 ± 40 mg/dL). HDL-C was lowest in AFLD (40 ± 10 mg/dL), intermediate in MASLD (45 ± 8 mg/dL), and highest in controls (55 ± 12 mg/dL). The atherogenic index (log[TG/HDL-C]) was significantly higher in MASLD (0.65 ± 0.18) than in AFLD (0.55 ± 0.21) and controls (0.35 ± 0.15; p < 0.001).

Conclusion: MASLD is associated with a more severe atherogenic lipid pattern marked by elevated TC, LDL-C, and TG, whereas AFLD features a more pronounced reduction in HDL-C. These distinct profiles suggest differing cardiovascular risk trajectories and support etiology-specific lipid management approaches.

## Introduction

Steatotic liver disease, comprising both alcoholic and metabolic-associated subtypes, has become a major contributor to cardiovascular illness and death [1]. As the primary organ regulating lipid homeostasis, the liver controls cholesterol synthesis, lipoprotein assembly, and triglyceride metabolism [2]. When hepatic steatosis develops, these regulatory systems become disrupted, producing characteristic lipid abnormalities that promote atherosclerotic progression [3].

Metabolic dysfunction-associated steatotic liver disease (MASLD) shares a close relationship with metabolic syndrome, and its associated dyslipidemia is actually one of the diagnostic criteria [4]. The classic lipid disturbance, i.e., elevated triglycerides (TG), low high-density lipoprotein cholesterol (HDL-C), and an abundance of small dense low-density lipoprotein (LDL) particles, establishes a strongly pro-atherogenic environment [5]. Alcohol-related fatty liver disease (AFLD), in contrast, has traditionally been linked to different lipid changes, notably a rise in high-density lipoprotein (HDL) with modest alcohol intake, but in real-world clinical settings, it frequently overlaps with metabolic abnormalities, making the lipid phenotype difficult to characterize [6].

Cardiovascular disease is the leading cause of death among MASLD patients, surpassing liver-related mortality [7]. Nevertheless, lipid management guidelines specifically tailored to liver disease are lacking, and fears about statin-induced hepatotoxicity often discourage aggressive treatment [8]. Understanding how lipid profiles differ between AFLD and MASLD could improve risk assessment and guide therapeutic decisions. Direct comparative studies are rare; most existing research examines these entities separately or concentrates on advanced cirrhosis [9]. The nature of dyslipidemia in early-stage liver disease, particularly in populations with distinct genetic and dietary characteristics such as those in India, needs further investigation [10].

Objectives

To compare conventional and advanced lipid parameters between AFLD, MASLD, and healthy controls.

Hypothesis

We hypothesize that MASLD will demonstrate a more atherogenic lipid profile characterized by higher triglycerides and LDL-cholesterol, while AFLD will show more pronounced HDL-cholesterol alterations.

## Materials and methods

Study design and ethical considerations

This analytical cross-sectional study was conducted from June 2024 to December 2024. Ethical approval was obtained (KVV/IEC/03/2024), and written informed consent was obtained from all participants. The study adhered to the STROBE (Strengthening the Reporting of Observational Studies in Epidemiology) guidelines for observational research.

Study population

Inclusion Criteria

Inclusion criteria included patients aged 18-80 years. The AFLD group consisted of individuals with alcohol consumption of 30 g/day or more for men or 20 g/day or more for women for five years or longer, along with hepatic steatosis confirmed on ultrasonography. The MASLD group included individuals with hepatic steatosis on ultrasonography, no alcohol consumption, and at least one metabolic risk factor. The control group comprised individuals with no hepatic steatosis, no alcohol consumption, and no components of metabolic syndrome.

Exclusion Criteria

Exclusion criteria included other chronic liver diseases (viral hepatitis B or C, autoimmune hepatitis, Wilson's disease, hemochromatosis); acute hepatic decompensation or hepatocellular carcinoma; malignancy or active inflammatory conditions; pregnancy or lactation; refusal to provide informed consent; and use of lipid-lowering medications within three months.

Laboratory methods

After a 12-hour overnight fast, venous blood was collected in plain vacutainers. Serum was separated within one hour and analyzed immediately. Lipid profile analysis was performed as follows: total cholesterol was measured using the CHOD-PAP (cholesterol oxidase peroxidase-aminoantipyrine-phenol) method; triglycerides were measured using the GPO-PAP (glycerol phosphate oxidase peroxidase-aminoantipyrine-phenol) method; HDL-cholesterol was measured using the direct homogeneous method; and LDL-cholesterol (LDL-C) was calculated using the Friedewald formula (valid for triglycerides less than 400 mg/dL). Non-HDL cholesterol was calculated as total cholesterol minus HDL-cholesterol. Calculated parameters included the atherogenic index (log of triglycerides divided by HDL-cholesterol), total cholesterol to HDL-cholesterol ratio, LDL-cholesterol to HDL-cholesterol ratio, and non-HDL-cholesterol to HDL-cholesterol ratio.

Cardiovascular risk assessment

Atherosclerotic cardiovascular disease (ASCVD) risk score was calculated using pooled cohort equations adapted for the Indian population [11]. The Framingham Risk Score was adapted using Asian-specific coefficients [12]. Atherogenic dyslipidemia was defined as the presence of all three: TG ≥150 mg/dL, HDL-C <40 mg/dL (men) or <50 mg/dL (women), and increased non-HDL-C.

Statistical analysis

Statistical calculations were performed using IBM SPSS Statistics version 25.0 (IBM Corp., Armonk, NY) and MedCalc version 20.0 (MedCalc Software Ltd, Ostend, Belgium). Continuous data are shown as mean ± standard deviation or median with interquartile range, depending on distribution normality. To compare groups, we used either one-way ANOVA, followed by Tukey's post-hoc test (for normally distributed data) or the Kruskal-Wallis test with Dunn's post-hoc correction (for non-normal distributions). Categorical variables were analyzed using the chi-square test. Correlation analyses employed Pearson's or Spearman's coefficients as appropriate. Independent predictors of lipid parameters were identified through multivariable linear regression. The ability of various lipid ratios to discriminate between disease groups was evaluated using receiver operating characteristic (ROC) curve analysis. A two-tailed p-value <0.05 was considered statistically significant.

All scales, scores, and indices used in this study were based on previously published sources. The ASCVD risk score was calculated using the pooled cohort equations as described by Grundy et al. [11]. The Framingham Risk Score was adapted using Asian-specific coefficients as described by D'Agostino et al. [12]. The definition of atherogenic dyslipidemia (TG ≥150 mg/dL, HDL-C <40 mg/dL in men or <50 mg/dL in women, and increased non-HDL-C) was based on the National Cholesterol Education Program Adult Treatment Panel III (NCEP ATP III) criteria. The definition of metabolic syndrome followed the Joint Interim Statement criteria [4]. All these scales and criteria are publicly available, and no permission was required for their use.

## Results

Baseline characteristics

Both patient groups (AFLD and MASLD) had significantly higher body mass index and blood pressure readings. Among the three groups, the MASLD group showed the highest proportion of individuals with diabetes (14 out of 30, 46.7%) and metabolic syndrome (13 out of 30, 43.3%).

Conventional lipid parameters

As summarized below, MASLD patients demonstrated the most adverse lipid pattern across nearly all parameters. Their mean total cholesterol (220 ± 25 mg/dL) was significantly elevated compared to both AFLD patients (200 ± 30 mg/dL, p = 0.008) and healthy controls (180 ± 20 mg/dL, p < 0.001). A similar pattern was observed for LDL-cholesterol, which was highest in the MASLD group (150 ± 20 mg/dL), intermediate in the AFLD group (130 ± 25 mg/dL), and lowest in controls (110 ± 15 mg/dL), with all intergroup differences reaching statistical significance (p < 0.001 for MASLD vs. controls and MASLD vs. AFLD).

HDL-cholesterol showed a different ordering: the AFLD group had the lowest values (40 ± 10 mg/dL), which were significantly lower than those in the MASLD group (45 ± 8 mg/dL, p = 0.02) and markedly lower than controls (55 ± 12 mg/dL, p < 0.001). Triglyceride levels were comparably elevated in both disease groups relative to controls (MASLD: 200 ± 50 mg/dL; AFLD: 180 ± 60 mg/dL; controls: 120 ± 40 mg/dL; both disease vs. control comparisons, p < 0.001) with no statistically significant difference between the two patient groups (p = 0.12) (Table 1).

**Table 1:** Atherogenic indices and cardiovascular risk scores. Data are presented as mean ± standard deviation (SD). Between-group comparisons were performed using one-way ANOVA with Tukey's post-hoc test for multiple comparisons. The ANOVA F-values (degrees of freedom = 2.87) were as follows: TC/HDL-C ratio (F = 20.1), LDL-C/HDL-C ratio (F = 18.7), non-HDL-C/HDL-C ratio (F = 19.4), atherogenic index (F = 21.3), ASCVD 10-year risk (F = 22.8), and Framingham Risk Score (F = 19.9). All p-values were <0.001. Statistical significance was set at p < 0.05. For post-hoc comparisons, p < 0.05, p < 0.01, and p < 0.001 are indicated where applicable. Abbreviations: AFLD, alcoholic fatty liver disease; MASLD, metabolic dysfunction-associated steatotic liver disease; TC, total cholesterol; HDL-C, high-density lipoprotein cholesterol; LDL-C, low-density lipoprotein cholesterol; ASCVD, atherosclerotic cardiovascular disease; SD, standard deviation. Note: Atherogenic index was calculated as log(triglycerides/HDL-C). ASCVD 10-year risk percentage was calculated using the pooled cohort equations adapted for the Indian population [11]. The Framingham Risk Score percentage was adapted using Asian-specific coefficients [12]. Higher scores indicate greater cardiovascular risk.

Parameter	AFLD	MASLD	Control	p-value
TC/HDL-C ratio	5.0 ± 1.2	4.9 ± 1.0	3.3 ± 0.8	<0.001
LDL-C/HDL-C ratio	3.3 ± 0.9	3.3 ± 0.8	2.0 ± 0.6	<0.001
Non-HDL-C/HDL-C	4.0 ± 1.1	3.9 ± 0.9	2.3 ± 0.7	<0.001
Atherogenic index	0.55 ± 0.21	0.65 ± 0.18	0.35 ± 0.15	<0.001
ASCVD 10-year risk (%)	8.5 ± 4.2	9.8 ± 3.8	3.2 ± 1.5	<0.001
Framingham Risk (%)	12.3 ± 5.1	14.2 ± 4.6	5.8 ± 2.3	<0.001

Advanced lipid indices and ratios

Data reveal that the atherogenic index, calculated as log(triglycerides/HDL-C), differed substantially across groups (F = 21.3, p < 0.001). MASLD patients had the highest value (0.65 ± 0.18), followed by AFLD patients (0.55 ± 0.21) and controls (0.35 ± 0.15). Post-hoc testing confirmed significant differences between MASLD and controls (p < 0.001), between MASLD and AFLD (p = 0.008), and between AFLD and controls (p < 0.001). The TC/HDL-C ratio was also markedly higher in both disease groups (MASLD: 4.9 ± 1.0; AFLD: 5.0 ± 1.2) compared to controls (3.3 ± 0.8; F = 20.1, p < 0.001) (Table 2).

**Table 2:** Comprehensive lipid profile analysis. Data are presented as mean ± standard deviation (SD). Between-group comparisons were performed using one-way ANOVA with Tukey's post-hoc test for multiple comparisons. The ANOVA F-values (degrees of freedom = 2.87) were as follows: total cholesterol (F = 18.4), LDL-C (F = 21.2), HDL-C (F = 16.7), triglycerides (F = 24.1), and non-HDL-C (F = 22.5). All p-values were <0.001 except where noted. Statistical significance was set at p < 0.05. * Post-hoc comparison symbols: M>C indicates MASLD significantly different from control; M>A indicates MASLD significantly different from AFLD; C>A indicates control significantly different from AFLD; A>C indicates AFLD significantly different from control. Abbreviations: AFLD, alcoholic fatty liver disease; MASLD, metabolic dysfunction-associated steatotic liver disease; LDL-C, low-density lipoprotein cholesterol; HDL-C, high-density lipoprotein cholesterol; Non-HDL-C, non-high-density lipoprotein cholesterol; SD, standard deviation; A, AFLD group; M, MASLD group; C, control group.

Parameter	AFLD (n = 30)	MASLD (n = 30)	Control (n = 30)	p-value	Post-hoc comparisons*
Total cholesterol (mg/dL)	200 ± 30	220 ± 25	180 ± 20	<0.001	M>C (p<0.001), M>A (p=0.008)
LDL-C (mg/dL)	130 ± 25	150 ± 20	110 ± 15	<0.001	M>C (p<0.001), M>A (p<0.001)
HDL-C (mg/dL)	40 ± 10	45 ± 8	55 ± 12	<0.001	C>A (p<0.001), C>M (p<0.001)
Triglycerides (mg/dL)	180 ± 60	200 ± 50	120 ± 40	<0.001	M>C (p<0.001), A>C (p<0.001)
Non-HDL-C (mg/dL)	160 ± 28	175 ± 22	125 ± 18	<0.001	M>C (p<0.001), A>C (p<0.001)

Pattern analysis

As summarized in Table 3, the MASLD-associated lipid pattern featured concurrent elevations in total cholesterol, LDL-cholesterol, and triglycerides alongside a moderate reduction in HDL-cholesterol. This triad is consistent with the dyslipidemia typically driven by insulin resistance. The AFLD-associated lipid pattern was characterized by a severe decline in HDL-cholesterol, accompanied by modest triglyceride elevation and relatively preserved LDL-cholesterol levels, reflecting alcohol-related disturbances in HDL metabolism and increased hepatic lipase activity.

**Table 3:** Pattern analysis of lipid abnormalities in AFLD vs. MASLD. Data are presented as N (%). Between-group comparisons were performed using the chi-square test. Statistical significance was set at p < 0.05. Abbreviations: AFLD, alcoholic fatty liver disease; MASLD, metabolic dysfunction-associated steatotic liver disease; TC, total cholesterol; LDL-C, low-density lipoprotein cholesterol; TG, triglycerides; HDL-C, high-density lipoprotein cholesterol.

Pattern feature	MASLD (n = 30)	AFLD (n = 30)	Test statistic	p-value
MASLD-associated lipid pattern (elevated TC, LDL-C, TG + moderate HDL-C reduction)	18 (60.0%)	8 (26.7%)	χ² = 6.8	0.009
AFLD-associated lipid pattern (severe HDL-C reduction + moderate TG elevation)	12 (40.0%)	22 (73.3%)	χ² = 6.8	0.009
Severe HDL-C reduction (<40 mg/dL)	12 (40.0%)	22 (73.3%)	χ² = 6.8	0.009

Multivariable regression analysis

Using stepwise multivariable linear regression (Table 4), we identified several independent predictors for key lipid abnormalities. For elevated triglycerides, MASLD diagnosis (β = 0.42) and elevated alanine aminotransferase (ALT) (β = 0.38) were significant. For low HDL-C, AFLD diagnosis (β = -0.45) and current smoking (β = -0.28) were significant (model R² = 0.54, p < 0.001). For elevated LDL-C, MASLD diagnosis (β = 0.48), diabetes (β = 0.32), and high dietary saturated fat (β = 0.25) were significant (model R² = 0.48, p < 0.001).

**Table 4:** Multivariable regression analysis – independent predictors of lipid abnormalities. Data are presented as β coefficients with 95% confidence intervals (CI). Stepwise multivariable linear regression was used. Statistical significance was set at p < 0.05. Abbreviations: AFLD, alcoholic fatty liver disease; MASLD, metabolic dysfunction-associated steatotic liver disease; ALT, alanine aminotransferase; HDL-C, high-density lipoprotein cholesterol; LDL-C, low-density lipoprotein cholesterol.

Outcome variable	Predictor	β (95% CI)	p-value	Model R²	Model F	Model p-value
Elevated triglycerides	MASLD diagnosis	0.42 (0.28-0.56)	<0.001	0.61	28.4	<0.001
	Elevated ALT (>40 U/L)	0.38 (0.24-0.52)	<0.001			
Low HDL-C	AFLD diagnosis	-0.45 (-0.62 to -0.28)	<0.001	0.54	22.1	<0.001
	Current smoking status	-0.28 (-0.47 to -0.09)	0.006			
Elevated LDL-C	MASLD diagnosis	0.48 (0.32-0.64)	<0.001	0.48	17.8	<0.001
	Diabetes mellitus	0.32 (0.13-0.51)	0.003			
	High dietary saturated fat (>10% calories)	0.25 (0.05-0.45)	0.018			

ROC analysis for disease discrimination

The atherogenic index (log[triglycerides/HDL-C]) showed strong discriminatory power in distinguishing between groups as detailed in Table 5. Differentiating MASLD from healthy controls yielded an area under the curve (AUC) of 0.92 (95% CI: 0.86-0.97, p < 0.001) with an optimal cutoff of >0.52 (sensitivity = 86.7%, specificity = 90.0%). Differentiating MASLD from AFLD yielded an AUC of 0.78 (95% CI: 0.68-0.87, p < 0.001) with a cutoff of >0.60 (sensitivity = 70.0%, specificity = 73.3%).

**Table 5:** ROC analysis – discriminative ability of atherogenic index (log[TG/HDL-C]). Abbreviations: ROC, receiver operating characteristic; AUC, area under the curve; CI, confidence interval; AFLD, alcoholic fatty liver disease; MASLD, metabolic dysfunction-associated steatotic liver disease; TG, triglycerides; HDL-C, high-density lipoprotein cholesterol.

Comparison group	AUC	95% CI	p-value	Optimal cut-off	Sensitivity (%)	Specificity (%)
MASLD vs. healthy controls	0.92	0.86-0.97	<0.001	>0.52	86.7	90.0
MASLD vs. AFLD	0.78	0.68-0.87	<0.001	>0.60	70.0	73.3
AFLD vs. healthy controls	0.82	0.74-0.90	<0.001	—	—	—

Cardiovascular risk stratification

As shown in Table 6, when applying the ASCVD risk score with a threshold of ≥7.5% to define high risk, we found that 17 of 30 MASLD patients (58%), 14 of 30 AFLD patients (48%), and only two of 30 controls (6%) fell into this category (χ² = 21.6, df = 2, p < 0.001). Very high-risk status (ASCVD ≥20%) was present in four MASLD patients (12%) and two AFLD patients (8%), but in none of the controls. Using the Framingham Risk Score with a moderate-risk cutoff of ≥10%, the proportions were as follows: MASLD = 16/30 (53.3%), AFLD = 12/30 (40.0%), and controls = 3/30 (10.0%) (χ² = 14.2, df = 2, p < 0.001).

**Table 6:** Cardiovascular risk stratification. Data are presented as N (%). Between-group comparisons were performed using the chi-square test. Statistical significance was set at p < 0.05. Abbreviations: AFLD, alcoholic fatty liver disease; MASLD, metabolic dysfunction-associated steatotic liver disease; ASCVD, atherosclerotic cardiovascular disease.

Risk category	MASLD (n = 30)	AFLD (n = 30)	Control (n = 30)	χ² (df = 2)	p-value
ASCVD risk score				21.6	<0.001
High-risk (≥7.5%)	17 (58%)	14 (48%)	2 (6%)		
Very high-risk (≥20%)	4 (12%)	2 (8%)	0 (0%)		
Framingham Risk Score				14.2	<0.001
Moderate-risk (≥10%)	16 (53.3%)	12 (40.0%)	3 (10.0%)		

## Discussion

The present study identifies clearly distinct patterns of atherogenic dyslipidemia when comparing AFLD and MASLD, with the latter showing a broader and more severe constellation of lipid abnormalities. These results highlight the liver's critical position in systemic lipid regulation and demonstrate how alcohol-induced toxicity versus metabolic dysfunction each produces different lipid signatures.

MASLD lipid phenotype

The simultaneous elevation of total cholesterol, LDL-C, and triglycerides, accompanied by reduced HDL-C in MASLD patients, is the hallmark of insulin-resistance-driven atherogenic dyslipidemia [13]. The underlying mechanisms include enhanced hepatic output of very low-density lipoprotein (VLDL) particles, diminished activity of lipoprotein lipase, and impaired HDL maturation [14]. Notably, the LDL-C level observed in our MASLD cohort (150 ± 20 mg/dL) was higher than figures typically reported from Western populations (130-140 mg/dL) [15], which may reflect genetic predispositions common in Indian populations, such as variants in the PNPLA3 and APOC3 genes [10]. The triglyceride elevation (200 ± 50 mg/dL) closely matches the "atherogenic triad" originally described by Frayn [5] in the context of metabolic syndrome.

AFLD lipid phenotype

In AFLD patients, we observed a striking reduction in HDL-C (40 ± 10 mg/dL) together with a moderate rise in triglycerides. This pattern may reflect the dual nature of alcohol's effects on lipid metabolism, whereas moderate intake can raise HDL acutely, chronic excessive consumption appears to accelerate HDL catabolism and compromise reverse cholesterol transport [16]. Additionally, alcohol is known to stimulate hepatic lipase activity, which reduces both the size and the number of HDL particles [17]. The degree of HDL-C reduction in our AFLD group was more severe than what has been documented in European cohorts (usually 45-55 mg/dL) [18], suggesting possible population-specific differences in alcohol metabolism, nutritional status, or concomitant tobacco use, the latter being present in 12 of 30 AFLD patients (40.0%) compared to only four of 30 MASLD patients (13.3%).

Comparison with the global literature

Our results are broadly consistent with prior reports but also extend them in several important ways. The total cholesterol level in our MASLD group (220 mg/dL) exceeded the range of 190-200 mg/dL typically seen in Western studies [17]; this disparity may be attributable to differences in dietary saturated fat intake and underlying genetic risk factors. The triglyceride elevation we observed is comparable to that reported in Asian cohorts from Hong Kong (190-210 mg/dL) [19], but our HDL-C values were lower (45 vs. 48-52 mg/dL), suggesting that the Indian population may face an even higher cardiovascular risk burden from MASLD. Furthermore, the HDL-C reduction in our AFLD patients was more pronounced than the pooled estimates from meta-analyses (40 vs. 45-50 mg/dL) [15], raising the possibility that population-specific factors, including alcohol metabolism genetics or coexisting micronutrient deficiencies (e.g., vitamin B12, folate), may modify the lipid response to chronic alcohol exposure.

Clinical implications

These findings carry several practical implications. First, patients with MASLD warrant a thorough cardiovascular risk assessment that includes a detailed lipid profile as an essential component. Second, the priority lipid targets may differ by etiology: in MASLD, triglycerides and LDL-C deserve particular attention, whereas in AFLD, raising HDL-C and controlling triglycerides should be emphasized. Third, serial lipid monitoring could serve as a useful surrogate marker for tracking liver disease progression. Fourth, statins have been proven safe in the setting of liver disease [20]; their underuse in this population represents a missed opportunity for cardiovascular protection. The American Association for the Study of Liver Diseases (AASLD) guidelines explicitly confirm that statins are safe in non-cirrhotic NAFLD/MASLD and even in compensated cirrhosis [8].

Several limitations of this study should be acknowledged. The cross-sectional design does not allow for causal inferences linking lipid abnormalities to liver disease etiology. Alcohol consumption history was obtained by self-report, which carries a risk of recall bias; however, we attempted to corroborate self-reports with gamma-glutamyl transferase (GGT) levels, which were elevated in 26 of 30 AFLD patients (86.7%). LDL-C values were calculated using the Friedewald equation, which loses accuracy when triglyceride levels exceed 400 mg/dL, a situation that did not occur in any of our participants. The sample size of 30 per group is relatively modest, limiting statistical power for subgroup analyses and precluding stratification by fibrosis stage. Direct measurements of small dense LDL particles, apolipoprotein B, or lipoprotein(a) were not performed; such measurements might have provided additional insights into atherogenic risk beyond conventional lipid parameters. The study was conducted at a single center in western India, which may limit generalizability to other ethnic or geographic populations. Dietary intake was assessed only through basic questions about saturated fat consumption rather than through detailed food frequency questionnaires. Finally, the cardiovascular risk scores (ASCVD, Framingham) we used were adapted for Indian populations, but the prospective validity of these adaptations has not been firmly established. Direct assessment of subclinical atherosclerosis, for example, via carotid intima-media thickness or coronary artery calcium scoring, would have provided more direct evidence of cardiovascular risk than surrogate lipid markers.

## Conclusions

In summary, MASLD and AFLD produce fundamentally different patterns of atherogenic dyslipidemia, which have important implications for cardiovascular risk management. MASLD is characterized by a broad range of lipid abnormalities typical of insulin resistance, whereas AFLD features a disproportionately severe reduction in HDL-C. These observations support the routine use of lipid profiling in all patients with steatotic liver disease, the adoption of etiology-specific lipid treatment strategies, aggressive cardiovascular risk reduction efforts in MASLD patients, and the development of integrated care models that address both hepatic and cardiovascular health. Early detection and appropriate management of dyslipidemia in these high-risk individuals may substantially lower the burden of cardiovascular morbidity and mortality.
